# Factors Affecting Recruitment and Attrition in Randomised Controlled Trials of Complementary and Alternative Medicine for Pregnancy-Related Issues

**DOI:** 10.1155/2016/6495410

**Published:** 2016-11-13

**Authors:** Ciara Close, Marlene Sinclair, Julie E. M. McCullough, Sarah Dianne Liddle, Ciara M. Hughes

**Affiliations:** ^1^Centre of Public Health, Queen's University, Belfast, Northern Ireland BT12 6BJ, Ireland; ^2^Institute of Nursing and Health Research, Ulster University, Shore Road, Newtownabbey, Co Antrim, Northern Ireland BT37 0QB, Ireland

## Abstract

*Background*. Randomised controlled trials (RCTs) investigating Complementary and Alternative Medicine (CAM) for pregnancy-related issues have encountered issues with recruitment and attrition. Little is known about the cause of these issues.* Methods*. Data was gathered from an antenatal CAM randomised controlled trial. During foetal anomaly appointments, women meeting inclusion criteria were invited to participate in the trial. Numbers of women invited and eligible were recorded. Reasons for noninterest were noted and analysed. Focus groups exploring trial experience of participants were also conducted.* Findings*. Of the 428 women invited to participate, 376 were eligible and just under a quarter participated. Reasons for nonparticipation included concerns about CAM and lack of interest in participation in research. Other factors negatively affecting recruitment included recruitment timing, competition for participants, limited support from staff, and inadequate trial promotion. Factors encouraging recruitment included being interested in research and seeking pain relief. Reasons for dropping out were time constraints, travel issues, work commitments, and pregnancy issues. Several women in the sham and usual care group dropped out due to dissatisfaction with treatment allocation.* Conclusion*. CAM researchers must explore problems encountered with recruitment and attrition so that evidence-based implementation strategies to address the issues can be developed.

## 1. Introduction

During pregnancy, women often suffer a range of complaints such as nausea, vomiting, heartburn, and low back and/or pelvic pain (LBPP), and many women turn to Complementary and Alternative Medicine (CAM) to alleviate these symptoms [1–4]. The percentage of women using CAM during pregnancy has been reported to be as high as 87% [5]. However, despite the high percentage of pregnant women using CAM, there are surprisingly few well-designed randomised controlled trials (RCTs) which have assessed the effectiveness of CAM interventions during pregnancy and many of the existing RCTs have encountered difficulties with recruitment and attrition [6–11].

CAM trials with pregnant women often have small sample sizes and this is likely related to issues with recruitment. Mollart [6] recruited just 96 pregnant women during a two-year period to an RCT investigating the effectiveness of reflexology for ankle oedema. In addition to this, in a pilot RCT investigating the effectiveness of chiropractic treatment and neuroemotional technique compared to exercise for pregnancy low back pain, researchers recruited just 57 pregnant women in 20 months. Kimber et al. [10] took 14 months to recruit 90 pregnant women to an RCT investigating massage for labour pain. Small sample sizes such as these affect the inferential capacity to make firm conclusions on the effectiveness of these CAM interventions for pregnancy-related issues. Recruitment of pregnant women into trials may not be an issue isolated to CAM trials, as other types of trials with pregnant women have struggled with recruitment. For example, recruitment rates to dietary and exercise trials with pregnant women have been reported to be between 19 and 24% [12–14].

High attrition of study participants has been an issue that has plagued many CAM trials with pregnant women. In RCTs, short term attrition rates of greater than 20% are considered unacceptable and may introduce bias into such trials [15]. Moreover, attrition rates greater than 30% in a specific arm of a trial indicate significant flaws exist in the study and the findings of such studies should be reviewed and interpreted with caution [15]. Overall attrition rates in CAM trials with pregnant women frequently exceed 20%. Kvorning et al. [11] investigated acupuncture for LBPP during pregnancy and reported an overall dropout rate of 28%. Similarly, Lund et al. [7] investigated acupuncture for pelvic pain in pregnancy and reported an overall dropout rate of 32%. However, other non-CAM trials with pregnant women have reported more acceptable overall attrition rates. For example, in an RCT investigating the effectiveness of the Bellybra support garment compared to a Tubigrip garment (control) for pregnancy back pain, attrition was much more acceptable at 18% [16]. Further to overall attrition rates, CAM trials with pregnant women have also experienced high attrition rates in comparison groups. For example, Wedenberg et al. [17] investigated the effectiveness of acupuncture for pregnancy LBPP compared to physiotherapy. There was a 40% attrition rate in the physiotherapy group and 0% attrition rate in the acupuncture group. The difficulty of blinding participants in CAM trials may be a factor associated with the high attrition issues discussed [18].

The suboptimal quality of available evidence for CAM use during pregnancy is concerning given the high percentage of pregnant women who report using these treatments. Therefore, improving the evidence base for CAM use during pregnancy needs to be prioritised, to equip maternity care professionals with the knowledge to provide evidence-based advice and support to pregnant women on the effectiveness and safety of using CAM interventions during pregnancy. In order to improve the evidence base for CAM, pressure has been put on research teams to conduct more RCTs in the area, as RCTs are recognised as the gold standard for assessing the effectiveness of interventions [19]. However, the suitability of using an RCT for assessing the effectiveness of CAM has been questioned given the many issues CAM RCTs have been shown to encounter in relation to recruitment, attrition, blinding, and disagreement on whether CAM treatments in RCTs should be standardised or should follow a pragmatic approach as used in clinical practice [20]. However, despite the questions raised around the suitability of RCTs to assess CAM effectiveness, it would seem that, given the current focus on evidence-informed practice, the emphasis on implementing RCTs to assess CAM effectiveness is set to continue. Therefore, exploring factors affecting pregnant women's participation and dropout in a CAM trial may aid better recruitment and reduce attrition to such studies in the future and ultimately may lead to a larger, more robust evidence base for CAM use during pregnancy which is desperately lacking.

The evidence base for the effectiveness of reflexology in pregnancy is particularly sparse and of dubious methodological quality. However, reflexology continues to be offered to pregnant women across the UK within maternity units and is one of the four most frequently used forms of CAM in these units [21]. Reflexology is promoted in these settings for managing a wide range of pregnancy-related issues including labour preparation and managing pregnancy-related symptoms such as back and pelvic pain. Given the use of reflexology in maternity settings in the absence of any robust evidence of effectiveness for pregnancy-related complaints, it was deemed important to explore the effectiveness of reflexology for pregnancy-related issues. The effectiveness of reflexology for pregnancy low back and pelvic pain was explored due to the fact that reflexology had been shown to be helpful for nonspecific low back pain in the general population [22].

*Aim.* This paper aims to explore the factors which affect recruitment and attrition in a pilot RCT investigating reflexology for pregnancy LBPP.

## 2. Methods

This paper presents secondary data from “The CAM in Pregnancy Trial,” (ISRCTN26607527) a pilot RCT investigating the effectiveness of reflexology for pregnancy LBPP as well as reporting on the findings from focus groups which were conducted with The CAM in Pregnancy Trial participants after their participation in the trial.

### 2.1. Ethical Approval

This research received full ethical approval from the Office of Research Ethics for Northern Ireland in July 2012.

### 2.2. RCT Description

The pilot RCT was designed and implemented aiming to determine if it was possible to conduct an RCT investigating the effectiveness of reflexology as an addition to usual care for pregnancy LBPP.

Women were randomly allocated to one of three groups, namely, reflexology plus usual care, footbath (as a sham intervention) plus usual care, or usual care only. Reflexology participants received six weeks of reflexology, footbath participants received six weeks of footbath treatments, and women in usual care received no additional treatment. The primary outcome measure was the frequency and intensity of their pain, which were assessed before and after the six-week study period. Secondary outcome measures were back specific function, mobility, and anxiety.

### 2.3. Midwives Briefing Session

Midwives were acting as the gatekeepers to the study population; therefore, it was considered essential that they were given an initial briefing about the study. In May 2012, a briefing session was given to the antenatal midwifery team at the maternity unit hosting the trial. The briefing session aimed to inform midwives about the trial, its purpose, and the inclusion and exclusion criteria (Table 1) before the trial began in July 2012. Midwives were provided with a health professional information sheet detailing information on all aspects of the study, and additional copies were placed in consultation rooms along with study posters to prompt midwives to discuss study participation with eligible women. Further to this, as and when new midwives joined the antenatal midwifery team, the researchers made efforts to introduce themselves and the study providing them with a health professional information sheet.

### 2.4. Recruitment

Over a 14-month period, pregnant women were invited to take part in the present trial at a routine foetal anomaly scan (normally 20–22-week gestation) in a large urban maternity unit, with approximately 4000 births annually. As mentioned, antenatal clinic midwives acted as the gatekeeper for the trial, introducing the study to potentially eligible pregnant women and asking for women's permission for a researcher to provide further verbal and written information on The CAM in Pregnancy Trial. If women were happy to receive further information on the trial from the researcher, the researcher joined the antenatal appointment. At this point, women who expressed an interest in participation were provided with a participant information sheet. The process of assessing eligibility and randomisation is described elsewhere [9]. Inclusion and exclusion criteria for The CAM in Pregnancy Trial are detailed in Table 1.

Women with a DVT, gestational diabetes, or placenta previa were excluded as complementary therapies are contraindicated in several health conditions. Women currently using CAM therapies were also excluded to remove possible confounding variables to investigate the effectiveness of reflexology for pregnancy-related low back and pelvic pain.

### 2.5. Recruitment Data

The research team gathered data on the number of women invited to participate and the number of women eligible. This information was analysed to provide percentage totals such as the percentage of eligible women that participated. The research team took written notes on reasons provided for nonparticipation. These reasons were compiled and analysed using thematic analysis.

### 2.6. Attrition Data

Data on attrition rates was generated from “The CAM in Pregnancy Trial.” Reasons for dropping out (where provided) were gathered and analysed using thematic analysis.

### 2.7. Focus Groups

Focus groups were conducted with women who had completed “The CAM in Pregnancy Trial.” The primary aim of these focus groups was to explore women's experience of low back and pelvic pain during pregnancy. However, a secondary aim of the focus groups was to explore women's experience of being part of “The CAM in Pregnancy Trial.” Women were specifically questioned about the positive and negative aspects of the trial from their perspective. Focus group findings were analysed using the Newell and Burnard framework for thematic analysis [23]. Further detail on the methodology and results from these focus groups is reported elsewhere [24].

## 3. Results

### 3.1. Recruitment

Recruitment took place between July 2012 and September 2013. Of the 428 primigravida women who were invited to take part in the pilot RCT, 52 did not meet the inclusion/exclusion criteria identified for this study. The major factor for exclusion from the pilot RCT was that the women were already participating in another research study at that time (*n* = 38). This had been identified as exclusion criteria because participating in two research studies would have placed additional burden on women, for example, in terms of their time and travel expenses. After exclusions, this left 376 potentially eligible women to participate in the pilot RCT.

Of the eligible women, 70% (*n* = 262) expressed an interest in participating in The CAM in Pregnancy Trial and therefore were provided with a participant information sheet. The remaining 30% (*n* = 114) of eligible women indicated from the outset that they did not wish to take part in The CAM in Pregnancy Trial or receive a participant information sheet. Of the eligible women who declined to participate at the point of recruitment, 32% (*n* = 37/114) provided a reason and this was mainly due to a lack of interest in research (Table 2). Unfortunately, as it was not part of the study protocol to ask women why they refused to participate in the trial, we were unable to obtain a reason for refusal to participate from the remaining 68% (*n* = 77/114) of eligible women who declined the invitation immediately after being invited to participate.

### 3.2. Attrition

64/90 (83%) of women randomised completed the six-week intervention period. A variety of reasons for dropping out of the six-week study period were provided. The most common being medical reasons, work commitments, and being unhappy with treatment allocation. Dropout rates were the highest in the footbath group and the lowest in the usual care only group (Table 3).

### 3.3. Focus Group Findings

The focus groups were attended by 14 women. Participants had a mean age of 33 and all had completed the six-week study period of The CAM in Pregnancy Trial; eight were in the reflexology group, two were in the usual care group, and four were in the footbath group. Most (13/14) of the participants had used CAM previously. Thematic analysis revealed three major themes in relation to women's experiences of the trial, each with two subthemes (Table 4).

The participant who provided a particular quote in the focus group will be coded as follows: participant identification number, age, and intervention group. The abbreviations in Table 5 will be used.

#### 3.3.1. Subtheme 1: Factors Negatively Affecting Recruitment

*Lack of Awareness and Support for the Trial from Maternity Staff*. Some women identified an issue that health professionals did not know that the study was happening and that other health professionals were not supportive of the trial:

*“My own community midwife was like…ah…I wish they had of told me about that…she was raging. She said I wish I had been told about that,” (PN14, 30 R)*

*“Whenever I was with my midwife they asked about the sticker on the front of my green folder and they had never heard of the study before,” (PN13, 27 FB)*

*“No offence to some of the midwives and I know some of them they were a bit blasé about it (The CAM in Pregnancy Trial),”  (*PN*3, 34  *R*)*

*“I think if that if they (Midwifery staff) were more…mmm more versed or had a wee bit more knowledge they might be a bit more proactive,” (PN3,34 R)*

*Poor Verbal and Visual Promotion of the Trial. *The visual and verbal promotion of the study may have been a factor for the slow recruitment to the trial:

*“I believe the posters were already up at this stage but I missed them…I must have missed them…they didn't catch my eye,” (P7, 32 R)*

*“If there was more detail in the poster,” (PN3, 34 R)*

*“It wasn't mentioned to me, at the 20-week scan and I mentioned to them about the pelvic pain,” (PN7, 32 R).*

#### 3.3.2. Subtheme 2: Factors Positively Affecting Recruitment

*Interested or Experienced in Research*. Women reported being motivated to participate in the trial due to having interest or experience in research:

*“Well I think research is important… I would be interested in research,” (PN2, 32 FB)*

*“I just thought research is really important… especially if there is loads of people in the future that it helps…I think you have go to start doing things like that,” (PN4, 30 UC)*

* “I am a research scientist…so I just wanted to be able to take part…to give back in some way…am I appreciate how hard it can be to get numbers,” (PN7, 32 R).*

*Desire for Symptom Relief*. While some women reported being motivated to participate in the trial for its research potential, other narratives indicated that the possibility of obtaining pain relief was the factor which motivated their participation. For some women, it was the nonpharmacological nature of CAM that attracted them to participate in the trial:

*“So as to see if it could be determined if reflexology was going to help…was going to be helpful for the pelvic pain..there was nothing helping me and there was nothing else on offer,” (PN11, 36, UC)*

*“It was probably a bit more selfish…I would have taken anything offered to me to be honest…I wasn't really thinking beyond that,” (PN12, 30, R)*

*“I am a great believer in holistic therapies… and I am not a big fan of taking pain killers…I think…I will be very honest I had a miscarriage before I had XXXX and didn't want to take any additional medication…in case it had an effect,” (PN3, 34 R)*

#### 3.3.3. Subtheme 3: Factors Negatively Affecting Attrition

*Maternity Staff Unblinding the Trial. *Comments suggested that unblinding of the active intervention had occurred. In several instances, the midwife who introduced the study to them indicated that reflexology was the treatment under investigation. Women reported on how midwives referred to the study as a “*reflexology study*” or “*free reflexology*”:

*“I was told it was free reflexology and that was how it was sold to me,” (PN10, 34 R LBPP)*

* “I think we could have got more information about each of treatment groups…I think I was led into believing it was reflexology,” (PN9, 35 FB)*

*“I was told it was reflexology and it would help your back pain the midwife didn't actually say to me at that point there are three options,” (PN8, 35 UC)*

*Footbath Was Not Helping Pain. *Attrition in the footbath may have been related to the fact that it was not helping with some women's pain:

*“I found it relaxing but not for pain,” (PN9, 35 FB)*

*“I think although it was great to get that time to myself…but it just wasn't helping my pelvic pain…and the thought of getting in and out of the car another time each week made me think what's the point if it's not helping my pain,” (PN2, 32 FB)*

## 4. Discussion

Recruitment was slower than anticipated for The CAM in Pregnancy Trial given that the trial was recruiting pregnant women with low back and pelvic pain, conditions highly prevalent in this population [25]. It was originally anticipated that recruitment of participants would take approximately one year. However, due to difficulties identifying suitable candidates, an additional two months was added to the recruitment period. Campbell et al. [26] explored rates of participation in RCTs and found that one-third of RCTs had to extend the recruitment time-frame to obtain the necessary sample size.

It is difficult to compare recruitment rates for The CAM in Pregnancy Trial and other CAM trials with pregnant women due to authors failing to report on the numbers of women invited to participate, the numbers of women assessed for eligibility, and the numbers of women that participated. There is a tendency in CAM trials to report only on the numbers of women that actually participated. However, when comparisons of recruitment rates are made with The CAM in Pregnancy Trial and with other CAM studies with pregnant women that reported detailed recruitment information, it appears that recruitment to The CAM in Pregnancy Trial (24%) was within normal ranges. For example, in a three-armed pilot RCT investigating massage therapy for reducing pain in labour, researchers recruited 21% of eligible women [10].

Recruitment rates to The CAM in Pregnancy Trial were also very similar to other intervention trials with pregnant women possibly suggesting that recruitment issues experienced in the present trial may be partly related to the population under investigation, a population often underrepresented in clinical trials. For example, in an RCT investigating the effectiveness of a dietary and physical activity intervention for preventing gestational diabetes, Luoto et al. [14] recruited 19% of eligible women. Currie et al. [13] highlighted that pregnant women are a challenging population to engage in public health interventions research, with issues such as work commitments and pregnancy conditions being common reasons for nonparticipation in studies. Work commitments and pregnancy conditions were issues in the present study in relation to attrition. Participation in the present trial initiated from approximately 27 weeks of gestation, which is the beginning of the third trimester of pregnancy, a time when pregnant women often suffer tiredness. Therefore, it is important to implement strategies where possible to reduce the burden of study participation on participants, for example, coordinating study appointments to take place at the same time as antenatal appointments.

The issues identified with recruitment and attrition in the present trial are a mixture of issues with pregnant women's participation in trials in general and pregnant women's participation to CAM trials. For example, recruitment timing is important for all studies with pregnant women, as insensitive or untimely invitations to participate in pregnancy trials may reduce recruitment rates. Recruitment to The CAM in Pregnancy Trial may have been too early in pregnancy, with recruitment taking place in the second trimester. LBPP is more prevalent in the third trimester of pregnancy and reaches peak intensity and interference at this time [27, 28]. Therefore, trial information provided to women not experiencing pain at this time would have been of little relevance to them. Women's decision to participate in a clinical trial during pregnancy is often guided by possible benefits to maternal and foetal health/well-being, but at 20–22 weeks many women do not have low back or pelvic pain so therefore there would be little incentive for them to participate [29, 30]. Prompting women again about participation in the study at an antenatal clinic appointment in the third trimester could have increased recruitment. Furthermore, recruitment of participants was at the foetal anomaly scan which may have been an issue. Some women need to have their foetal anomaly scan repeated due to the foetus being in a suboptimal position, which can be distressing. Therefore, these women may not have taken on board the information on The CAM in Pregnancy Trial provided at the time.

The recruitment of those not currently using CAM presents an issue in relation to significantly reducing the pool of eligible women particularly as CAM treatments are very popular with pregnant women for managing pain conditions like pregnancy LBPP [31]. It is highly possible that many of the women invited to participate may have been using CAM and thus may not have stepped forward to take part after being informed by the midwife and/or researcher that this was an exclusion to trial participation. Furthermore, the exclusion of current CAM users could be seen as being possibly bias, as exclusion of current CAM users may have meant that participants had little knowledge or interest in CAM. However, in an attempt to minimise this possible bias, the research team ensured that previous CAM experience was not exclusion criteria.

Another issue which may affect recruitment of pregnant women to trials in general is competition for study participants from other trials being conducted simultaneously. The APP trial was also recruiting healthy first-time mothers at the same time as the present trial but was recruiting women earlier at their booking appointment at eight—14 weeks of gestation. This meant that, at the recruitment point for The CAM in Pregnancy Trial, some women were already participating in the APP trial which reduced the number of eligible participants. Competition for participants has previously been reported as a barrier to effective recruitment into research studies [32, 33]. Better planning of recruitment timing and the use of a multicentred approach to recruitment may have improved recruitment rates of the trial.

Focus group findings indicated that not all eligible women were informed about the study indicating that discussions about the trial with eligible women were ad hoc. The reasons why midwives did not inform all eligible women about the study are unclear. Nonrecruitment of potentially eligible women by gatekeepers such as maternity health professionals would appear to be an issue more generally for trials with pregnant women. Research has shown that these health professionals frequently report lack of time, lack of awareness of ongoing trials, and lack of knowledge of trial inclusion criteria as reasons for nonrecruitment of pregnant women into research studies [32–35]. Midwives often change department in maternity units and so newer midwives to departments where trials are being conducted may not be aware that studies are taking place. This may be due to their absence at study information sessions or due to study information being put in inaccessible locations, for example, under other paperwork as identified by Stuart et al. [34, 35]. This also happened in the current study where trial information sheets for health professionals were put in a locked cabinet and so were not easily accessible to staff. Maternity staff may need regular briefing sessions about ongoing research projects to ensure that all staff, including those new to the department, are fully aware of current studies and their inclusion/exclusion criteria. Regular briefing sessions may also provide an opportunity for researchers to provide details on recruitment rates which may encourage staff to be more proactive with introducing the study to eligible women. Focus group comments from women also suggested that there was a lack of support from some midwifery staff when women mentioned that they were taking part in The CAM in Pregnancy Trial. Provision of regular briefing sessions on CAM trials with pregnant women to maternity professionals may be of even greater importance than other trials with pregnant women given that midwives attitudes towards CAM use in pregnancy can vary from some midwives being overenthusiastic about its benefits to others being totally dismissive of its potential for effectiveness [21, 36]. Therefore, it is possible that midwives owning personal attitudes towards CAM may have affected recruitment rates.

Another issue which may have affected recruitment was the visual promotion of the trial; and this may be an issue for poor recruitment rates to pregnancy trials in general. Women discussed during the focus group how they did not see the poster in the hospital and others thought that the poster did not give an accurate reflection of what the pilot RCT involved. During The CAM in Pregnancy Trial, only one A3 poster advertising the study was on display for most of the 14-month recruitment period. Originally, there were 12 study posters on display throughout the antenatal clinic waiting area but for unknown reasons an obstetrician removed all but one poster shortly after the trial began recruiting. It could be speculated that this may be related to the personal views of this obstetrician. Research with maternity professionals has shown that midwives and obstetricians have contrasting opinions on the effectiveness of CAM [37]. A survey by Gaffney and Smith [37] reported that 88 (65%) midwives agreed that alternative therapies are effective in encouraging the body's natural healing ability, yet only 13 (19%) obstetricians agreed with this statement and few would recommend CAM. In contrast to this, obstetricians appear more supportive of medications during pregnancy as opposed to alternative medicines for pain. For example, a survey of obstetricians found that 80% felt paracetamol was appropriate to recommend during pregnancy for dental pain [38]. Better use of social media such as Facebook and Twitter may have been useful for increasing recruitment rates. Shere et al. [39] found that the addition of these forms of social media increased recruitment of pregnant women into an RCT investigating folic acid by 12-fold compared to traditional sources of promotion such as posters, leaflets, and brochures.

A specific issue related to recruiting pregnant women to CAM trials is* “safety concerns,”* which is understandable given that the majority of CAM is not provided by the NHS but rather is available from private individuals/ organisations, which may make pregnant women dubious about accessing such treatments if they are not provided within routine health service provision [40]. Tooher et al. [32, 33] suggest that perception of risk is an important issue when pregnant women are making decisions about trial participation. If the pregnant women's perception of risk is too high, they will ultimately refuse to take part in an RCT [29]. Research has shown that pregnant women generally put more priority on the health of their baby rather than their own health [30]. While only eight women actually reported concern about the safety of reflexology during pregnancy as a reason for declining the invite to participate in the present trial; many other women questioned the researchers at the initial recruitment about the safety of reflexology in relation to its ability to induce labour. Concerns about reflexology are unsurprising given the lack of clarity about the role of reflexology in labour induction and the fact that midwives recommend the use of reflexology for induction of labour [41, 42]. In these circumstances, it is important that maternity staff make it clear to women that there is no sound scientific evidence that reflexology stimulates labour and that the few studies that have found an impact of reflexology on expediting labour are of low quality and employed specific reflexology techniques which would never be performed in studies assessing reflexology for other pregnancy-related issues.

During recruitment, it was observed that women frequently looked towards the midwife for reassurance that reflexology was safe to use during pregnancy. The level of reassurance provided by midwives in these instances varied. Women's concerns about using reflexology during pregnancy along with limited reassurance about the safety of using reflexology during pregnancy from some midwives could have been a contributory factor in the slow recruitment rates. Adopting a universal phrase about the safety of CAM in the pregnant population may have been helpful for ensuring women's confidence in participation in a CAM trial. Indeed, several RCTs investigating reflexology have been conducted on pregnant populations and no adverse effects have been reported. Maternity professionals must draw on high quality RCT or systematic review evidence (if available) when providing a statement about the safety of reflexology or other CAM treatments if questioned by prospective CAM trial participants [41–43].

In spite of the fact that some of the findings of this paper are focused on factors which negatively affect recruitment of pregnant women into trials, there were some findings from focus groups which highlighted factors which may positively facilitate recruitment into a CAM trials with pregnant women. These included desire for pain relief in general and specifically pain relief that was nonpharmacological. These findings concur with research conducted by Mohanna [30] who explored decision-making processes of pregnant women regarding participation in trials and also research by Wang et al. [31] who explored women's preferences regarding treatment for pregnancy low back pain. Researchers recruiting pregnant women into CAM trials may wish to make it clear that CAM is a nonpharmacological treatment as well as referring to available evidence related to its effectiveness and safety for pregnancy-related symptoms for conditions like pregnancy LBPP.

Attrition rates in the sham group were another major issue in The CAM in Pregnancy Trial, with a 50% attrition rate in this group. High attrition rates in the footbath group indicate significant flaws in the study design [12]. The high attrition rates may be explained by women's reported hopes of getting randomised to the reflexology group. Several women in the usual care and footbath groups reported that the reason why they dropped out of the trial was that they had not been randomised to receive reflexology. Attrition rates have been found to be problematic in previous CAM trials with pregnant women. Mollart [6] had similar issues with dropouts in a single blind RCT investigating the effectiveness of two types of reflexology for ankle oedema compared with a control group who received no reflexology but were rather instructed to rest on an examination couch for the same length of time as reflexology treatments. Seven of the 69 women randomised to the rest control group dropped out immediately after randomisation, three of whom stated their reason for dropping out was that “rest doesn't work”. The possible reason for high attrition rates in the present study and other similar CAM trials may be due to blinding issues. If participants are aware of which treatment is the treatment of interest for the study, it is understandable that many would not wish to continue with the study if they realise that they have been randomised to a sham intervention.

Blinding in the present study may not have been successful in many instances. Women's comments suggested that they knew that reflexology was the treatment of interest. The unblinding of the study by midwives is understandable as reflexology is one of the CAM therapies most frequently offered in maternity units in the UK [21]. Furthermore, some of midwives in the antenatal clinic where The CAM in Pregnancy Trial was conducted were reflexology-trained themselves and it is probable that these midwives may have been overenthusiastic about the reflexology aspect of the research, inadvertently placing more emphasis on the reflexology when introducing the research to women.

### 4.1. Limitations

Only a small number of the women invited to participate in The CAM in Pregnancy Trial actually provided reasons for refusing to take part which reduces the external validity of the current results. This was a limitation in the research design as it was not part of the study protocol to ask women for a reason for their nonparticipation and as such the women who provided reasons for nonparticipation may differ from those who did not, thus raising questions about the representativeness of the sample.

## 5. Conclusion

General barriers identified to pregnant women's participation in trials included timing of recruitment, competition for participants from other trials, and inadequate trial promotion. Specific barriers to pregnant women's participation in CAM trials included women's perception of the risk of participation and limited support for maternity staff. Poor study blinding was likely to be the major reason for the high attrition rates for the sham treatment group in the present trial. Researchers wishing to recruit pregnant women to trials and specifically CAM trials need to consider the barriers to recruitment and factors associated with high attrition identified in the present study and identify appropriate strategies to overcome these commonly occurring issues in CAM trials with pregnant women. Otherwise, CAM trials with pregnant women will continue to be of poor quality and the evidence base for CAM during pregnancy will remain suboptimal.

## Figures and Tables

**Table 1:** Inclusion and exclusion criteria for The *CAM in Pregnancy Trial*.

Inclusion criteria	Exclusion criteria
First-time pregnant women	Women pregnant with more than one baby
Being ≥18 years old	Smokers
Presence of low back pain and/or pelvic pain	Women with neurological diseases
Being in 26–29 weeks of gestation	Deep Vein Thrombosis (DVT) sufferers
Ability to understand written and verbal English	Fungal foot infections or verrucae
	Current use of CAM therapies
	Placenta Previa grade 3 or grade 4
	Already participating in a research study
	Any serious spinal pathology, for example, cancer, cauda equina, infection in the spine
	Previous road traffic accident
	Previous surgery to the hip, back, or pelvic region
	Inflammatory arthritis, for example, rheumatoid arthritis
	Diabetes/gestational diabetes
	Cardiac related problems
	Women whom the midwife deems unable to participate

**Table 2:** Reasons provided for declining invitation to participate in The CAM in Pregnancy Trial at the point of recruitment.

Reason	Number of women (*n* = 37)
Not interested in taking part in research	21 (57%)
Concerned about using CAM in pregnancy	8 (22%)
Disliking having feet touched	4 (11%)
Travel issues	2 (5%)
Pain so bad and inability to make weekly appointments for the study	2 (5%)

**Table 3:** Reasons provided by participants for dropping out of The CAM in Pregnancy Trial.

Reflexology dropouts (*n* = 6)	Footbath dropouts (*n* = 15)	Usual care dropouts (*n* = 5)
1: preeclampsia 1: travel issues 2: no longer have pain 1: verruca 1: unknown	1: preeclampsia 3: work commitments 3: unhappy with treatment allocation (wanted reflexology) 1: travel issues 1: personal reasons 1: feeling too tired 5: unknown	1: preeclampsia 1: unhappy with treatment allocation (wanted reflexology) 3: unknown

**Table 4:** Themes describing women's experiences of participating in The CAM in Pregnancy Trial.

Main theme	Subtheme
Factors negatively affecting recruitment	(i) Lack of awareness and support for the trial from maternity staff (ii) Poor visual and verbal promotion of the trial

Factors positively affecting recruitment	(i) Interested or experienced research (ii) Desire for pain relief

Factors negatively affecting attrition	(i) Maternity staff unblinding the trial (ii) Footbath not helping with pain

**Table 5:** Abbreviations for focus group quotations.

Term	Abbreviation
Participant number	PN
Reflexology	R
Footbath	FB
Usual care	UC
